# Quantitative modeling of responses to chronic ionizing radiation exposure using targeted and non-targeted effects

**DOI:** 10.1371/journal.pone.0176476

**Published:** 2017-04-25

**Authors:** Igor Shuryak

**Affiliations:** Center for Radiological Research, Columbia University, New York, NY, United States of America; ENEA Centro Ricerche Casaccia, ITALY

## Abstract

The biological effects of chronic ionizing radiation exposure can be difficult to study, but important to understand in order to protect the health of occupationally-exposed persons and victims of radiological accidents or malicious events. They include targeted effects (TE) caused by ionizations within/close to nuclear DNA, and non-targeted effects (NTE) caused by damage to other cell structures and/or activation of stress-signaling pathways in distant cells. Data on radiation damage in animal populations exposed over multiple generations to wide ranges of dose rates after the Chernobyl nuclear-power-plant accident are very useful for enhancing our understanding of these processes. We used a mechanistically-motivated mathematical model which includes TE and NTE to analyze a large published data set on chromosomal aberrations in pond snail (*Lymnaea stagnalis*) embryos collected over 16 years from water bodies contaminated by Chernobyl fallout, and from control locations. The fraction of embryo cells with aberrations increased dramatically (>10-fold) and non-linearly over a dose rate range of 0.03–420 μGy/h (0.00026–3.7 Gy/year). NTE were very important for describing the non-linearity of this radiation response: the TE-only model (without NTE) performed dramatically worse than the TE+NTE model. NTE were predicted to reach ½ of maximal intensity at 2.5 μGy/h (0.022 Gy/year) and to contribute >90% to the radiation response slope at dose rates <11 μGy/h (0.1 Gy/year). Internally-incorporated ^90^Sr was possibly more effective per unit dose than other radionuclides. The radiation response shape for chromosomal aberrations in snail embryos was consistent with data for a different endpoint: the fraction of young amoebocytes in adult snail haemolymph. Therefore, radiation may affect different snail life stages by similar mechanisms. The importance of NTE in our model-based analysis suggests that the search for modulators of NTE-related signaling pathways could be a promising strategy for mitigating the deleterious effects of chronic irradiation.

## Introduction

Chronic ionizing radiation exposure can affect human health and ecosystem functioning [1–3]. For example, nuclear power industry workers, miners, pilots, astronauts, and some medical professionals are faced with protracted occupational exposures to ionizing radiation [4–6]. Nuclear power plant accidents (e.g. Chernobyl, Fukushima) dispersed radionuclides over wide areas inhabited by humans and wildlife. Potential stored radioactive waste leakage events and terrorist attacks involving radioactive materials (e.g. radiological dispersal device, so called “dirty bomb”) can also result in large-scale radioactive contamination. However, despite its importance, chronic radiation has been under-studied because protraction of irradiation over time scales of days to years can be difficult due to resource and time constraints [7, 8].

In general, reduction of the radiation dose rate reduces the deleterious effects per unit dose [9, 10]. In other words, a dose delivered over a few hours often causes less toxicity (e.g. clonogenic cell death) than the same dose delivered over a few minutes. This sparing effect is believed to occur because radiation-induced damage repair can proceed throughout the exposure period. The repair rate counteracts the damage induction rate, reducing the amount of simultaneously present damage (e.g. DNA double strand breaks) and the probability of damage interactions (e.g. chromosomal aberrations caused by incorrect DSB rejoining).

However, if the exposure is protracted over very long times such as multiple cell cycles or multiple organism generations, additional phenomena come into play: metabolism, reproduction, developmental pathways and cell proliferation/differentiation programs must operate under continuously stressful conditions, and susceptibility to radiation effects often changes with the organism’s age/stage. In other words, the biological responses to acute and chronic irradiations can be qualitatively different because radiation acts as a transient stressor in the first scenario and as a continuous one in the second [11, 12].

The ability of a given organism to resist one type of radiation stress does not necessarily correlate with its ability to resist the other. Examples of poor correlations between resistance to acute and chronic irradiation have been found in prokaryotes [13–15] and in mammalian cell lines [16]. Factors such as radiation-induced division delay (e.g. the duration of temporary proliferation arrest caused by the DNA damage response) and cell cycle redistribution (e.g. accumulation of irradiated cells in sensitive or resistant phases of the cell cycle) can play an important role in these phenomena. At the level of multicellular organisms, reproductive performance and embryonic development are often much more radiosensitive endpoints than adult mortality [17, 18]. Consequently, continuous chronic irradiation could permanently compromise a population’s self-renewal capacity and therefore could be much more deleterious to a population than a single acute exposure.

Ionizing radiation acts on cells through multiple mechanisms. Ionizations in or very close to DNA (e.g. in the first hydration layer) can produce DSBs and other forms of DNA damage which can kill the cell or potentially transform it into a pre-malignant state [10, 19]. These phenomena can for convenience be called targeted effects (TE). Importantly, damage to nuclear DNA and to other cell structures (e.g. mitochondria) as well as radiation-induced changes in the redox balance (e.g. production of reactive oxygen species, ROS) can activate intra-cellular and inter-cellular signaling pathways [20]. Those cells which have not themselves been traversed by ionizing radiation tracks, but have received signals from cells which have been traversed, can experience non targeted effects (NTE) of radiation. Such effects, often called bystander effects, include altered differentiation, proliferation and migration, altered redox status (e.g. persistent oxidative stress) and gene expression, cell death (e.g. apoptosis), as well as various forms of genomic damage and instability (e.g. increased frequencies of micronuclei, mutagenesis, chromosomal aberrations) [20–24]. Adaptive responses (e.g. upregulation of antioxidant defense mechanisms and DNA repair) can also be induced by NTE [25, 26]. Therefore, NTE can maintain a whole organ (or even an entire organism) in an altered (e.g. stressed) state even when not all the cells in the organ have been traversed by ionizing tracks.

The signaling pathways involved in NTE are complex and incompletely understood. However, their consequences can be quantitatively modeled in a simple and tractable manner by using the following assumptions: (1) Irradiated cells “activate” nearby cells in an “on-off” (binary) manner. (2) Activated cells accumulate DNA damage (e.g. chromosomal aberrations) at an elevated rate. Eventually they can revert to the background state, but this process may be very slow in some cases (e.g. require years-decades). (3) If the radiation dose is protracted, cells remain activated longer. The result is an increased yield of damaged cells–an inverse dose-rate effect, where chronic irradiation becomes more effective per unit dose than acute irradiation. Such effects have been observed after exposure to densely ionizing radiation, e.g. for lung cancer in radon-exposed miners [27–29]. (4) Activation-induced damage adds to the damage produced by direct traversal of targets by radiation, i.e. both TE and NTE contribute to the radiation response.

We previously applied models based on these assumptions to *in vitro* and *in vivo* data [21, 22]. Here we used the same approach to analyze a large published data set on chromosomal aberrations in pond snail (*Lymnaea stagnalis*) embryos collected over 16 years (1998–2014) from 6 water bodies contaminated by Chernobyl fallout, and from 2 control locations with background radiation levels [30, 31]. *L*. *stagnalis* is a useful model system because it is a common aquatic invertebrate throughout the contaminated area and in adjacent areas with background radiation [30]. It has a high reproduction rate, and both adult individuals and egg masses can be easily collected from studied water bodies [30, 32]. Consequently, Gudkov et al. [31] were able to analyze a total of 307,540 snail embryo cells for the presence of chromosomal aberrations. They also performed detailed radiation dosimetry calculations at all studied locations, estimating total dose rates and contributions of various radionuclides [30, 31]. The fraction of snail embryo cells with ≥1 chromosomal aberrations increased strongly (>10-fold) over a dose rate range of 0.03–420 μGy/h (0.00026–3.7 Gy/year).

The resulting data set, which is very large and combines dose rate estimates with radiation-induced damage measurements (chromosomal aberration frequencies), contains valuable quantitative information about chronic radiation effects over multiple generations and under natural (rather than laboratory) conditions. In addition to the embryo data, Gudkov et al. performed hematological studies on the haemolymph of adult *L*. *stagnalis*, measuring young amoebocytes, dead and phagocytic cells [31]. These studies provide data for a different endpoint, allowing radiation response shapes for different endpoints in embryos and adults to be compared. For example, similarity of response shapes for both endpoints can be interpreted as evidence for similarity of the biological effects of radiation on different life stages of *L*. *stagnalis*.

We analyzed the data using our model to: (1) determine whether or not a simple modeling approach could describe the main features of the responses to chronic irradiation in animal populations under natural conditions; (2) quantify the magnitude of potential NTE contributions to these radiation responses. The latter point is important because NTE, which are driven by intra- and inter-cellular signaling pathways, can potentially be modulated by exogenously administered chemical agents. If the NTE contribution to the effects of low dose rate chronic irradiation is large, then development of such agents could be a promising strategy for protecting human health from chronic radiation exposures.

## Materials and methods

### Data sets

The main features of the data set on the radiation responses of pond snails (*Lymnaea stagnalis*) collected from water bodies with various amounts of radioactive contamination after the Chernobyl nuclear power plant accident, which was published by Gudkov et al. [30, 31], were described above. There were two studied water bodies (Opechen and Vyrlitsa lakes) where snails were exposed to near-background radiation dose rates of 0.03–0.04 μGy/h (2.6–3.5×10^−4^ Gy/year) and six water bodies (Glubokoye, Dalyokoye and Azbuchin lakes, Yanovsky crawl, Pripyat and Uzh rivers) where dose rates were much higher: 0.2–420 μGy/h (1.8×10^−3^–3.68 Gy/year) [31]. Data were collected over 16 years, from 1998 until 2014.

Gudkov et al. [31] scored the following types of chromosomal aberrations were scored in anaphase and telophase snail embryo cells: single fragments; twin fragments; single bridges; single bridges with fragments; single bridges with two fragments; twin bridges; twin bridges with single fragments; twin bridges with two fragments; and multiple aberrations (more than four abnormalities per cell). For these studies, snail egg masses were preserved in Carnoy's fluid. For staining of cytological preparations, 1 g orcein dissolved in 45 mL of boiling acetic acid was used, and 55 mL of distilled water were added. Each sample consisted of 10 eggs in a watch glass containing 5mL of orcein, maintained at 4 degrees C for 24 h [31].

The observed fractions of snail embryo cells with ≥1 chromosomal aberrations at each studied water body and sampling year were presented graphically in Fig 2 of Gudkov et al. [31]. We digitized these data using the GetData Graph Digitizer software (http://www.getdata-graph-digitizer.com/). We assumed that the point estimates from Gudkov et al. [31] represented mean values, and that error bars represented standard errors. Using this information and the total number of analyzed snail embryo cells reported by Gudkov et al. [31], we reconstructed the estimated number of analyzed cells (*N*) for each water body and year. This reconstruction was based on the assumption that the fraction of cells with aberrations was a binomial proportion, so that *N* = *F*_*obs*_ ×(1 –*F*_*obs*_)/*SE*_*obs*_^2^, where *F*_*obs*_ is the observed fraction of cells with aberrations and *SE*_*obs*_ is the standard error. The results of this data set reconstruction are listed in S1 Data. The second analyzed data set on the fraction of young amoebocytes in adult snail haemolymph was reconstructed by digitizing the data from Fig 7 of Gudkov et al. [31]. Error bars were not provided for these data, and therefore we estimated the numbers of analyzed cells from each water body by dividing the total number of analyzed cells by the number of studied water bodies.

### Radiation dose response model

The main assumptions about the role of NTE in our radiation dose response model were described above and in previous publications [21, 22]. Briefly, we assumed that traversal of a cell by radiation can cause the release of NTE-mediating signals. The signal concentration reaches a steady-state equilibrium value in the target organ(s), which is proportional to the radiation dose rate. The equilibrium signal concentration determines the equilibrium probability (*P*_*e*_) for a cell in the organ(s) to be in an “activated” state. This probability is described by the following equation, where *R* is the excess radiation dose rate (total dose rate minus the natural background) and *q* is the excess dose rate at which 50% of all susceptible cells are activated (Table 1):

$$P_{e}=1/[1+q/R]$$ (1)

Details about the derivation of Eq 1 are provided in reference [21]. At very low dose rates *P*_*e*_ is close to zero, and at very high dose rates it approaches 1. The dependence of *P*_*e*_ on *R* is non-linear.

**Table 1:** The meanings of model parameters.

Model parameter	Meaning
*bac*	Background mean number of chromosomal aberrations per cell
*a*	Excess aberrations per cell from TE (year/Gy)
*b*	Excess aberrations per cell from maximally-intense NTE
*q*	Dose rate at which NTE reach ½ of maximal intensity (Gy/year)
*c*	Decrease of radioactive contamination effects over time (year^-1^)

We assumed that NTE contribute to the steady-state mean number of chromosomal aberrations per snail embryo cell (*M*) by the term *b*×*P*_*e*_, where *b* is the yield of excess aberrations from maximally-intense NTE (Table 1). TE of course also contribute to *M*, by the term *a*×*R*, where *a* is the adjustable parameter (Table 1). Both the TE and NTE terms are added to the background number of aberrations per cell (*bac*), which occur under natural background radiation exposure.

When these terms are combined, the mean number of aberrations per cell *M*(*R*) at excess dose rate *R* is described by the following equation:

$$M(R)=bac+(a\times R+b/[1+q/R])\times exp[-c\times (T-T_{0})]$$ (2)

Here the term exp[-*c*×(*T*–*T*_*0*_)] represents a decrease in radiation-induced effects over time: *T*_0_ is the year when observations began (1998 in the data set analyzed here [31]), *T* is the year of interest, and *c* is an adjustable parameter (Table 1). This exponential decrease is intended to represent the combined effects of the following phenomena: physical decay of the dominant radionuclides, reduction of bioavailable radionuclide concentrations in the studied water bodies, and possible reduction of radiation effect severity due to organismal adaptation [32].

The “slope” of the radiation response could be estimated at any selected dose rate by differentiating Eq 2 over dose rate. The solution for this derivative, *dM*(*R*)/*dR*, is:

$$\frac{dM(R)}{dR}=(a+b\times q/[R^{2}\times {\left(1+q/R\right)}^{2}])\times exp[-c\times (T-T_{0})]$$ (3)

### Dose rate estimates

Gudkov et al. [31] did not specify during what year(s) of the study the radiation dose rates were estimated. We assumed that this was done during the first year of study– 1998. This assumption about the calendar year to which dose rate estimates are assigned should not affect the conclusions of our analysis (e.g. the shape of the radiation response relationship), as long as the dose rates were estimated at the same time in all studied water bodies.

In an earlier publication, Gudkov et al. [30] provided not only total dose rates, but also reported the contributions of specific radionuclides and radionuclide groups. Specifically, they listed: (a) the dose rate from internally-incorporated ^137^Cs, ^90^Sr, ^238^Pu, ^239+240^Pu, ^241^Am; (b) the dose rate from ^137^Cs and ^90^Sr in the water; and (c) the dose rate from external γ-rays [30]. This information is potentially useful for assessing whether or not certain radionuclides had higher biological effectiveness per unit dose in this system, than others. For example, α-particle emitters (e.g. Am and Pu isotopes) may be more biologically effective than γ-ray emitters (e.g. ^137^Cs) because α-particles and γ-rays differ in energy deposition patterns and complexity of induced DNA damage [19, 33, 34].

We used the data from Table 1 in Gudkov et al. [30] to extract the following values for each studied water body: (1) the minimum and maximum dose rates from each (*i*-th) radionuclide or radionuclide group (a-c above), called *R*_*Min*_(*i*) and *R*_*Max*_(*i*), respectively; (2) the corresponding minimum and maximum total dose rates from all radionuclides combined, called *R*_*totMin*_ and *R*_*totMax*_, respectively. *R*_*Min*_(*i*) and *R*_*Max*_(*i*) were then be converted to fractional contributions, *R*_*fracMin*_(*i*) and *R*_*fracMax*_(*i*), as follows:

$$R_{fracMin}(i)=\frac{R_{Min}(i)}{R_{totMax}};\;R_{fracMax}(i)=min[1,\frac{R_{Max}(i)}{R_{totMin}}]$$ (4)

We assumed that *R*_*fracMin*_(*i*) and *R*_*fracMax*_(*i*) were applicable to the total dose rates reported in the subsequent publication from Gudkov et al. [31]. To assess potential differences in biological effectiveness of different radionuclides, we performed separate analyses where the following radionuclides were allowed to have higher biological effectiveness than other radiation sources (e.g. external γ-rays): (A) α-particle emitting Am and Pu isotopes, (B) all internally-incorporated radionuclides, (C) internally-incorporated ^90^Sr, or (D) total ^90^Sr (internal and external). The excess radiation dose rate (R) for these analyses was calculated as follows, where *R*_*bacMin*_ and *R*_*bacMax*_ were minimum and maximum values for the background dose rate (in the two reference water bodies) and *WF*(*i*) was the weighting factor for the *i*-th radionuclide group (A-D):

$$R_{gen}={\left[(R_{totMin}-R_{bacMin})\times {(R}_{totMax}-R_{bacMax})\right]}^{\frac{1}{2}};\;R_{fracGM}(i)={\left(R_{fracMin}(i){\times R}_{fracMax}(i)\right)}^{\frac{1}{2}};\;R={[WF(i)\times R}_{fracGM}(i)+(1-R_{fracGM}(i))]\times R_{gen}$$ (5)

Here we used geometric means to produce point estimates for the relevant dose rates. In situations where *R*_*fracMin*_(*i*) and *R*_*fracMax*_(*i*) were unknown (not reported in reference [30]), they were set to 10^−9^ and 1, respectively.

As described below in the subsection about model parameter uncertainties, we performed sensitivity calculations to assess how these simplifying assumptions (which were motivated by limitations of the published data from references [30, 31]) affected the results. These calculations involved random perturbations of dose rate estimates: instead of geometric means, point estimates of dose rates were generated by random draws from the uniform distribution bounded by the relevant minimum and maximum values. For example, *R*_*fracGM*_(*i*) was a uniformly-distributed random number between *R*_*fracMin*_(*i*) and *R*_*fracMax*_(*i*).

The values of *R* estimated using Eq (5), or by Monte Carlo (MC) simulation (described below), were inserted into Eqs (2 and 3) to obtain model predictions. For the simple case where the weighting factor for the *i*-th radionuclide group *WF*(*i*) was equal to 1, *R* reduced to *R*_*gen*_. In other situations, when *WF*(*i*) was >1, *R* became larger than *R*_*gen*_.

### Assumptions about error distributions

The error distribution around the mean *M*(*R*) from Eq 2 was assumed to be Poisson. This is a reasonable assumption for chromosomal aberrations induced by sparsely-ionizing radiation [35, 36]. According to the Poisson distribution, the predicted fraction of cells with ≥1 chromosomal aberrations (*F*(*R*)) is described by the following equation, where *M*(*R*) is taken from Eq (2):

F(R)=1−exp⁡[−M(R)] (6)

The same model structure (Eq 2) was used on the other analyzed data set (adult snail haemolymph composition) by assuming that the fraction of young amoebocytes is equal to 1 –*s*×*F*(*R*), where *s* is an adjustable parameter.

We also considered a scenario with a negative binomial (NB) error distribution. This distribution is more appropriate for chromosomal aberrations induced by densely-ionizing radiation (e.g. α-particles), where the variance can be considerably larger than the mean [35, 37]. For ease of interpretation, the NB distribution was parametrized as follows, where *P*_*NB*_*(k)* is the probability of observing *k* aberrations in a cell, Γ is the Gamma function, and *r* is the “overdispersion” parameter:

Q=M(R)+1/r,

$$P_{NB}(k)={\left(1/[r\times Q]\right)}^{1/r}\times {\left(M(R)/Q\right)}^{k}\times \Gamma (k+1/r)/[\Gamma (1/r)\times k!]$$ (7)

Using this parametrization, the variance is described by the convenient expression *M*(*R*) + *r*×*M*(*R*)^2^. Consequently, if *r* approaches 0, there is no overdispersion and the variance and mean are equal, as in the Poisson distribution. On the other hand, if *r* > 0, then the variance becomes greater than the mean and the ratio of variance to mean increases as the mean increases.

According to the NB distribution, the predicted fraction of cells with ≥1 chromosomal aberrations (*F*_*NB*_(*R*)) is described by the following equation:

$$F_{NB}(R)=1-{\left[1+r\times M(R)\right]}^{-1/r}$$ (8)

The data set analyzed here [31] contained only the observed fraction of cells with ≥1 chromosomal aberrations, but not the full distribution of aberration frequencies. Consequently, parameter *r* could not be determined from the data. We therefore used Eq (6), based on the Poisson distribution, as the default assumption for our analysis, but also performed exploratory calculations using Eq (8) with artificially assigned values of *r* (0.1, 0.5, 1.0 or 2.0). These exploratory calculations were intended to assess the sensitivity of analysis results to the potential presence of overdispersion in the data. They showed that varying parameter *r* affected the best-fit model parameters, but these effects were not dramatic and they did not change any of the major conclusions described below. Therefore, we focus on the results of using Eq (6) rather than Eq (8) in the subsequent sections.

### Model fitting procedure

The dose response model predicted the fraction of cells with ≥1 chromosomal aberrations: *F*(*R*) from Eq (6) or *F*_*NB*_(*R*) from Eq (8). To fit these predictions to the data, i.e. to the observed fractions of cells with ≥1 aberrations *F*_*obs*_(*R*), we assumed that each analyzed snail embryo cell represents an independent Bernoulli trial which can result in 2 outcomes: either the cell has 0 aberrations or ≥1 aberrations. We believe that this assumption is a reasonable approximation for the available data taken from reference [31]. Using it, we constructed the following Binomial distribution for the probability *P* of observing *K* cells with ≥1 aberrations out of *N* analyzed cells, where *F*(*R*) or *F*_*NB*_(*R*) is the model-predicted fraction of cells with ≥1 aberrations:

$$P=N!\times {F(R)}^{K}\times {\left(1-F(R)\right)}^{N-K}/[K!\times (N-K)!]$$ (9)

In other words, *N* and *K* represent the observed data (from a specific water body, dose rate and year of observation), *K/N* is the fraction of cells with aberrations reported in reference [31], and *F*(*R*) are corresponding model predictions. The goal is to find parameter values *bac*, *a*, *b*, *q* and *c* that maximize the product of likelihoods (values of *P*) for all the data (for all water bodies and years of observation). This task can be accomplished more conveniently by maximizing the log likelihood (*LL*), which is defined as *LL* = ln[*P*]. The solution for *LL* is:

LL=K×ln⁡[F(R)]+(N−K)×ln⁡[1−F(R)]+ln⁡[1/(N−K)!]+ln⁡[N!]−ln⁡[K!] (10)

The terms ln[1/(*N*–*K*)!] + ln[*N*!]–ln[*K*!] do not contain *F*(*R*) and, therefore, have no role in determining the best-fit values of parameters *bac*, *a*, *b*, *q* and *c*. They can be omitted for convenience.

We maximized the sum of log likelihoods (*LL* from Eq 10) for all data using the sequential quadratic programming (SQP) algorithm [38] implemented in Maple 2016® software. To increase the probability that a global rather than a local maximum was found for the *LL* function, we performed the procedure 100 times with different randomly-selected initial parameter values. The best results from these 100 repeats, i.e. the parameter values from the particular repeat which produced the largest *LL*, were recorded.

### Model parameter uncertainties

Uncertainties (95% confidence intervals, CI) for each model parameter (*bac*, *a*, *b*, *q* and *c*) were estimated by profile likelihood [39], which is based on the asymptotic X^2^ behavior of the log likelihood distribution. In addition, we further explored model parameter uncertainties by sensitivity calculations. They consisted of MC simulation which included random variability in dose rate estimates and aberration counts. Specifically, we produced 10,000 synthetic data sets based on the real data set. In each synthetic data set: (1) the dose rate was a uniformly-distributed random number between the minimum and maximum dose rate values reported in references [30, 31], as described above (Eq 5); (2) the number of analyzed snail embryo cells was a Poisson-distributed random number with the mean set to the observed value (*N*, estimated as described above); (3) the number of embryo cells with ≥1 aberrations was a Poisson-distributed random number with the mean set to the observed value (*K*, estimated as described above). The model was fitted to each synthetic data set using Eq (10), and best-fit parameter values were recorded. Parameter uncertainties (95% CIs) were estimated using the 2.5^th^ and 97.5^th^ percentiles of the distribution of values for each parameter across the 10,000 simulations. The goal of these analyses was to estimate model parameter uncertainties on unperturbed data (by profile likelihood) and to test the sensitivity of these estimates to random fluctuations in the data (by MC simulation).

### Quantification of the NTE contribution to the radiation response

The fractional NTE contribution (*NTE*_*c*_) to the radiation response slope (Eq 3) can be estimated by the expression *NTE*_*c*_ = 1 –*dM*(*R*, *b* = 0)/*dM*(*R*), where *dM*(*R*) is the slope from Eq (3) and *dM*(*R*, *b* = 0) is the slope with NTE parameter *b* set to zero (leaving only the TE contribution). For convenience, in this calculation one can use *T* = *T*_*0*_. The solution for *NTE*_*c*_ is:

$${NTE}_{c}=b\times q/[b\times q+a\times {\left(q+R\right)}^{2}]$$ (11)

At very low dose rates *NTE*_*c*_ approaches *b*/[*b* + *a*×*q*], and at high dose rates it approaches 0. In other words, the NTE contribution is highest at low dose rates where few cells are traversed by ionizing tracks and TE are therefore small. At very high dose rates the NTE contribution declines and TE start to dominate.

To estimate the uncertainties of *NTE*_*c*_, we used MC simulation to generate random parameter values in the vicinity of the best-fit values. The simulation continued until 10,000 parameter combinations which produced model predictions that fell within the 95% confidence critical contour of the log likelihood function were recorded. In other words, we accumulated model parameter combinations which produced fits almost as good as the best fit. These parameter combinations were substituted into Eq (11). In this manner, a distribution of *NTE*_*c*_ values was created at any selected dose rate. The minimum and maximum values from the distribution were used to approximate the 95% CIs of *NTE*_*c*_.

### Information theoretic (IT) model selection

An additional method to estimate the role of NTE in describing the data involved IT model selection. A reduced TE-only model was created by substituting *b* = *q* = 0 into Eq (2). This reduced model was fitted to the data by the same log likelihood maximization procedure as the full (TE+NTE model, Eq 2). The maximized log likelihoods for the full and reduced models were compared by converting them into sample size corrected Akaike information criterion (AICc) values [40, 41]. In this manner, models were ranked by relative support from the data, taking into account sample size and number of adjustable parameters.

The relative likelihood of the M-th model, called the evidence ratio (ER_M_), can be expressed as follows, where AICc_min_ is the lowest AICc value generated by the set of models being compared:

ERM=exp⁡[−12ΔAICcM],whereΔAICcM=AICcM−AICcmin (12)

The normalized evidence ratio, i.e. the evidence ratio for the tested model divided by the sum of the evidence ratios for all the models being compared, is another useful quantity which is called the Akaike weight, *W*_M_. It represents the probability that the M-th model would be considered the best-supported model (among those tested) upon repeated sampling of the data. The formula for the Akaike weight is:

$$W_{M}={ER}_{M}/\sum_{M}{ER}_{M}$$ (13)

As a hypothetical example, suppose that the TE-only model has an Akaike weight of 0.8, which means that the full TE+NTE model’s weight is 1–0.8 = 0.2. This result implies that the presence of NTE cannot be ruled out with high confidence, but that the simpler TE-only model has better support from the data. On the other hand, if the TE-only model has an Akaike weight of only 0.01, one can conclude that NTE are very important for describing the data because the model which does not include them performs poorly, compared with the model which does include NTE.

In addition to the TE+NTE and TE-only models, we used the IT metrics ΔAICc and W to compare the performances of various other model versions. Specifically, an NTE-only model was generated by setting *a* = 0 in Eq (2). A time-dependent dose rate (TDR) model was generated as follows by assuming that the decrease in radiation-induced effects over time, i.e. the term exp[-*c*×(*T*–*T*_*0*_)], applies directly to the dose rate, rather than to the biological effects:

$$M(R)=bac+(a\times R\times exp[-c\times (T-T_{0})]+b/[1+q/(R\times \exp\left[-c\times (T-T_{0})\right])])$$ (14)

Multiple other model versions were produced by substituting dose rete estimates produced using weighting factors *WF*(*i*) for specific radionuclides, which were described earlier, into each of these model structures (TE+NTE, TE-only, NTE-only, TDR). For each version, Poisson and NB error distributions were analyzed separately.

## Results

**Fig 1:**
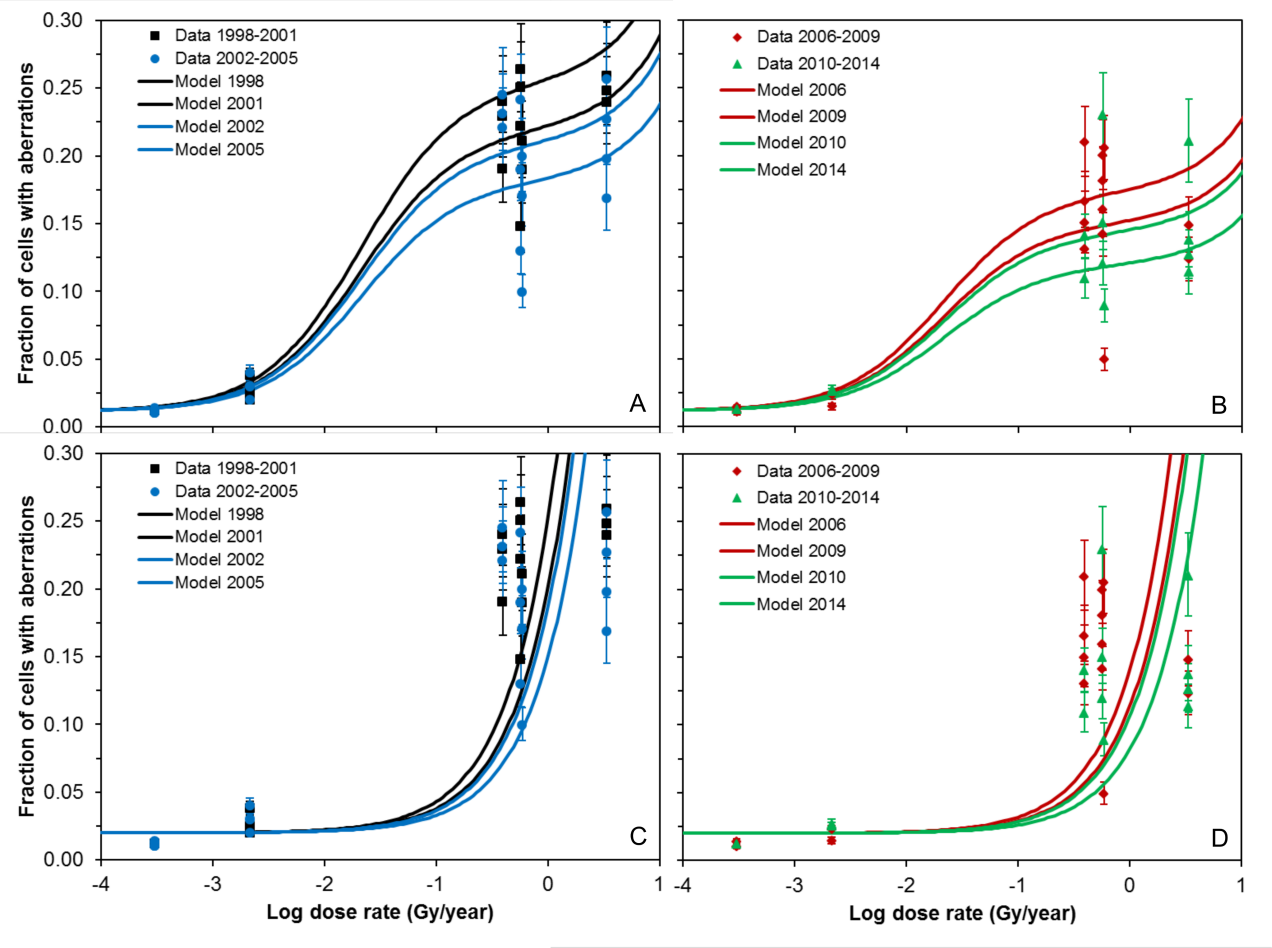
Comparison of data (symbols) and best-fit predictions (curves) for the standard model which uses both TE and NTE (panels A-B) or the TE-only model which does not use NTE (panels C-D). Best-fit parameter values for both model versions are provided in Table 2. The focus here is the shape of the response to radiation dose rate. Error bars represent 95% CIs calculated using the score confidence interval method for binomial proportions [42].

The data on chromosomal aberrations in snail embryos exposed to different radiation dose rates [31] are shown in Fig 1 (symbols). In reference water bodies (Opechen and Vyrlitsa lakes) with background radiation dose rates the fraction of snail embryo cells with chromosomal aberrations was generally low. In lightly contaminated water bodies (Pripyat river and Uzh river), where the dose rate was about 10-fold above background, the fraction of cells with aberrations was moderately increased (about 1.5-fold) above the background value. In highly contaminated water bodies (Globokoye, Dalyokoye and Azbuchin lakes, Yanovsky crawl), where dose rates were about 1,000–10,000-fold above background, the fraction of cells with aberrations was strongly increased (>10-fold) above the background value.

The radiation response shape was clearly non-linear. Specifically, the response showed some increase between uncontaminated (background) locations and lightly contaminated locations (dose rates between 2.6–3.5×10^−4^ and 1.8–2.6×10^−3^ Gy/year), but the steepest rate of increase occurred between lightly and highly contaminated locations (dose rates between 1.8–2.6×10^−3^ and 0.31–0.51 Gy/year). The increase became less steep at high contamination levels (dose rates between 0.31–0.51 and 3.07–3.68 Gy/year) [31]. The approximately 10-fold variation in dose rates between different highly contaminated water bodies (e.g. between Globokoye and Dalyokoye lakes) resulted in a much less than 10-fold variation in aberration frequency.

The best-fit model curves adequately described the main features of these data (Fig 1A and 1B). Specifically, the model was consistent with the non-linearity of the radiation response shape. Such non-linearity was described mainly by NTE: when the NTE parameters *b* and *q* were set to zero, the resulting TE-only model had a dramatically lower fit quality than the standard TE+NTE model (Fig 1C and 1D): its performance was worse by 5123 AICc units (Table 2).

**Table 2:** Best-fit model parameter values and comparison of model fit qualities. The results for four model versions (standard = TE + NTE, Eq (2); TE-only = NTE parameters *b* and *q* were set to zero; NTE-only = TE parameter *a* was set to zero; TDR = time-dependent dose rate, Eq 14) are arranged in columns. ΔAICc and Akaike weight are information theoretic metrics of relative model performance: the model with the highest support from the data among all tested models has the lowest ΔAICc and the highest weight. Details are discussed in the main text.

Model assumptions	Standard	TE-only	NTE-only	TDR
**ΔAICc**	0.0	5123.2	8.1	300.8
**Akaike weight**	0.983	0.000	0.017	0.000
**Parameter *bac***	0.0117	0.0200	0.0117	0.0121
** **95% CIs	0.0116	0.0199	0.0116	0.0116
** **	0.0120	0.0206	0.0120	0.0126
**Parameter *a*** (year/Gy)	0.0080	0.2313	0	0.0292
** **95% CIs	0.0031	0.2280	0	0.0201
** **	0.0130	0.2428	0	0.0393
**Parameter *b***	0.242	0	0.251	0.153
** **95% CIs	0.236	0	0.244	0.150
** **	0.247	0	0.256	0.158
**Parameter *q*** (Gy/year)	0.0216	0	0.0227	0.0110
** **95% CIs	0.0203	0	0.0213	0.0105
** **	0.0236	0	0.0248	0.0122
**Parameter *c*** (year^-1^)	0.0506	0.0828	0.0499	0.0998
** **95% CIs	0.0465	0.0796	0.0459	0.0769
** **	0.0551	0.0852	0.0544	0.1202

The best-fit model curves (Fig 1) adequately described the main features of these data. Specifically, the model was consistent with the non-linearity of the radiation response shape. Such non-linearity was described mainly by the NTE component of the model. When the NTE parameters *b* and *q* were set to zero, the resulting TE-only model had a dramatically lower fit quality than the standard model: its performance was worse by 5123 AICc units (Table 2).

In other words, a TE-only model generated a linear radiation response which was inconsistent with the shape of the observed data (Fig 1C and 1D). In contrast, when the TE parameter *a* was set to zero, the resulting NTE-only model performed only 8 AICc units worse than the standard model (Table 2). These patterns remained unchanged when the error distribution for the number of chromosomal aberrations per cell was changed from Poisson to negative binomial with various degrees of overdispersion, as described in Materials and Methods. The overdispersed distributions produced slightly worse model fits than the Poison ones, but the relative ranking of models remained the same. These results suggested that a decent fit to the data could be generated only by including NTE in the model.

The dose rate at which NTE were predicted to reach ½ of maximal intensity (parameter *q*) was approximately 0.022 Gy/year, i.e. 2.5 μGy/h (Table 2). Such a dose rate is intermediate between values observed in lightly and highly contaminated water bodies [30, 31]. The mean number of excess chromosomal aberrations per cell from maximally-intense NTE (parameter *b*) was approximately 0.24 (Table 2). The radiation response coefficient from TE (parameter *a*) was about 0.008 year/Gy, i.e. 7.0×10^−5^ h/μGy (Table 2).

**Fig 2:**
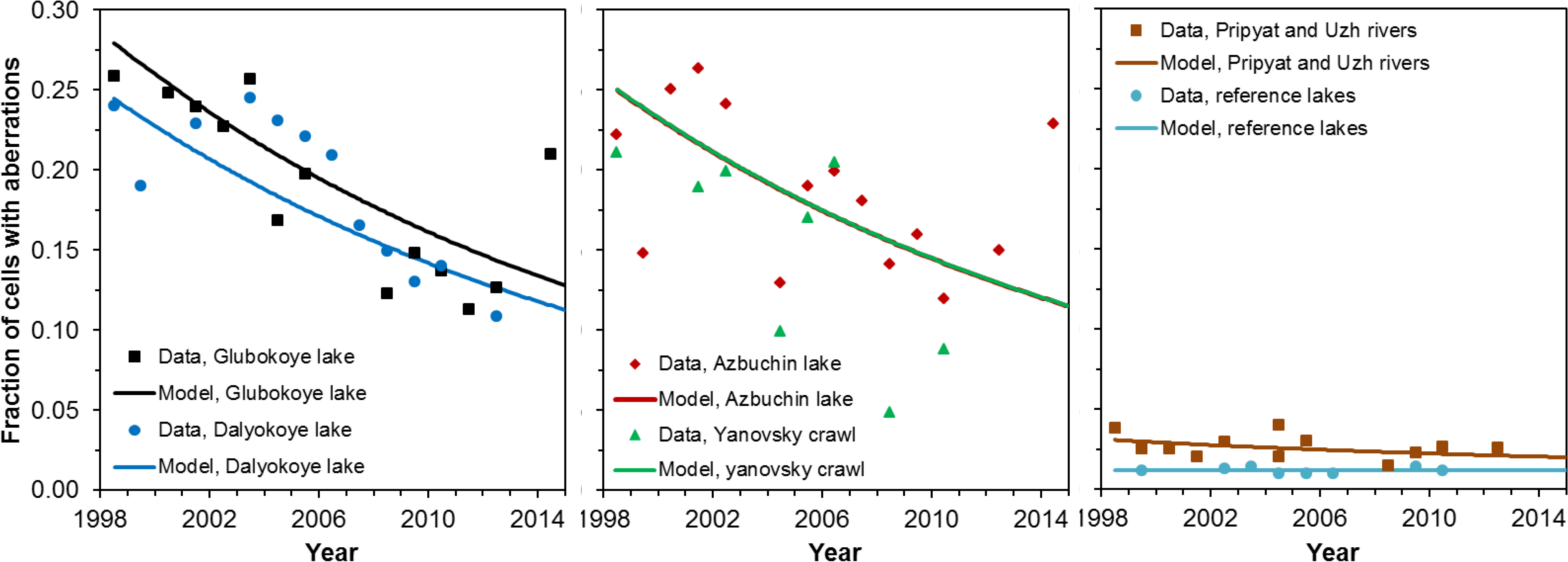
Comparison of data (symbols) and best-fit predictions (curves) for the standard model. The focus here is the time dependence of radiation effects. In this and the following figures, error bars on the data points are not shown to make visualization of the data more convenient. They are provided in Fig 1.

The model assumption that the radiation-induced excess aberration frequency decreases at an exponential rate (parameter *c*) with time after the Chernobyl accident in all water bodies was consistent with the data (Fig 2). In contrast, an alternative assumption that the excess dose rate, rather than excess aberration frequency, decreased with time had very poor support from the data: such a model (labeled TDR, Table 2) performed worse than the standard model by 301 AICc units.

The best-fit rate of decrease for the radiation response (parameter *c*) was about 0.05 year^-1^ (Table 2), i.e. the half-life was about 14 years. This estimate is approximately 2-fold smaller than the physical half-lives of the main radionuclide contaminants ^137^Cs and ^90^Sr. This discrepancy suggests that reduction of long-term radiation effects in the studied snails may occur not only due to physical decay of radionuclides, but also due to chemical/biological processes that reduce radionuclide concentrations in the water bodies and in snail tissues [32].

**Fig 3:**
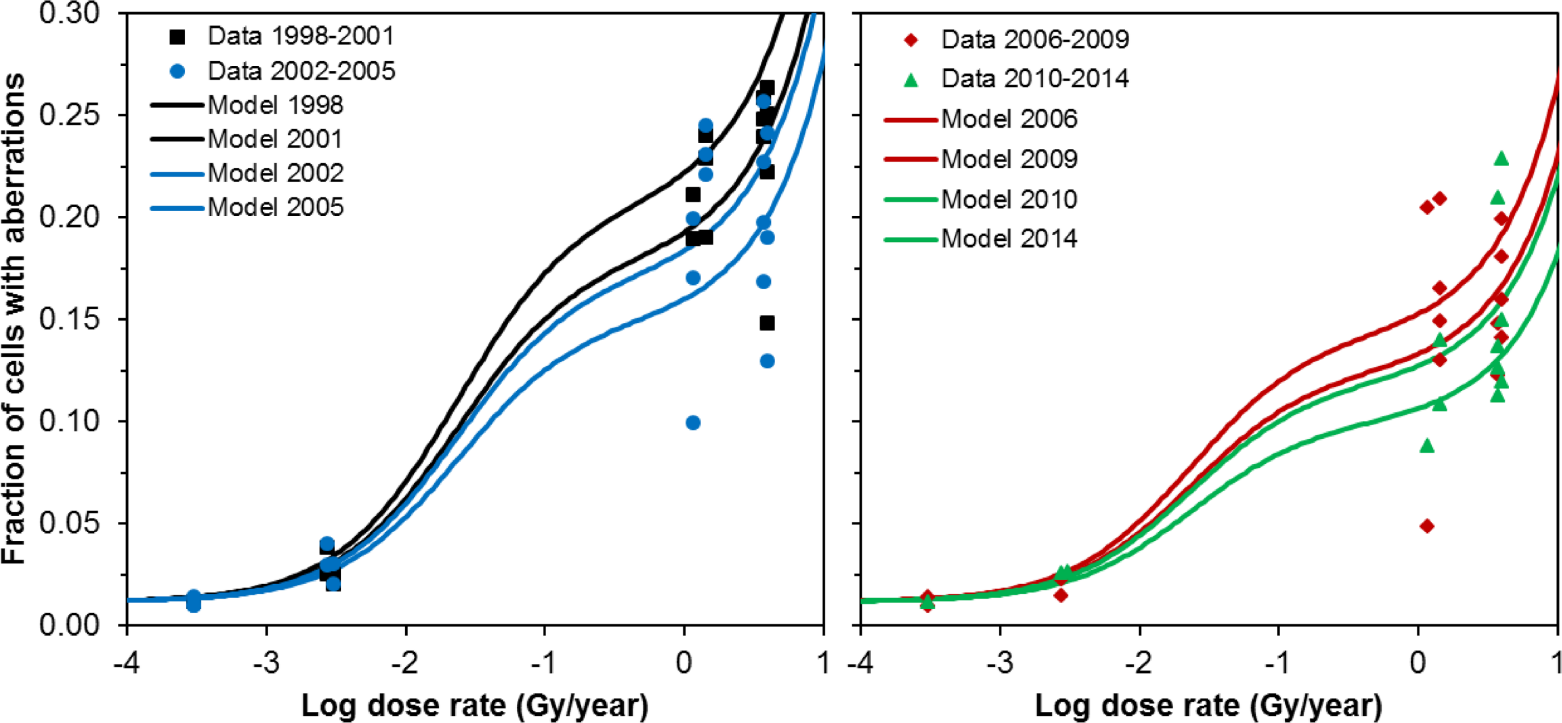
Comparison of data (symbols) and best-fit predictions (curves) for the internal Sr model. In this model the dose rate component from internally-incorporated ^90^Sr was weighted by a factor of 25. Details are described in the main text.

The contributions of different radionuclides (e.g. internally-accumulated ^90^Sr or α-particle emitters such as Pu and Am isotopes) to the total dose rate experienced by snails differed substantially among different water bodies [30]. As described in the Materials and Methods section, we used this information to investigate potential differences in biological effectiveness between radionuclides by assigning weighting factors >1 to the dose rate contributions of these radionuclides. In other words, in the standard model the dose rates from external and internally-accumulated radionuclides were treated equally and added together, but in these calculations selected radionuclides or radionuclide groups were allowed to be more biologically effective than the rest. The highest support among all tested model variants was achieved when the dose rate component from internally-incorporated ^90^Sr was weighted by a factor of 25 (95% CI: 13, 46) (Fig 3). This formalism, called the internal Sr model for convenience, fit the data by 48.4 AICc units better than the standard model. However, we cannot exclude the possibility that this difference in fit quality could have been amplified by the procedure used to reconstruct data from limited published information [30, 31].

**Fig 4:**
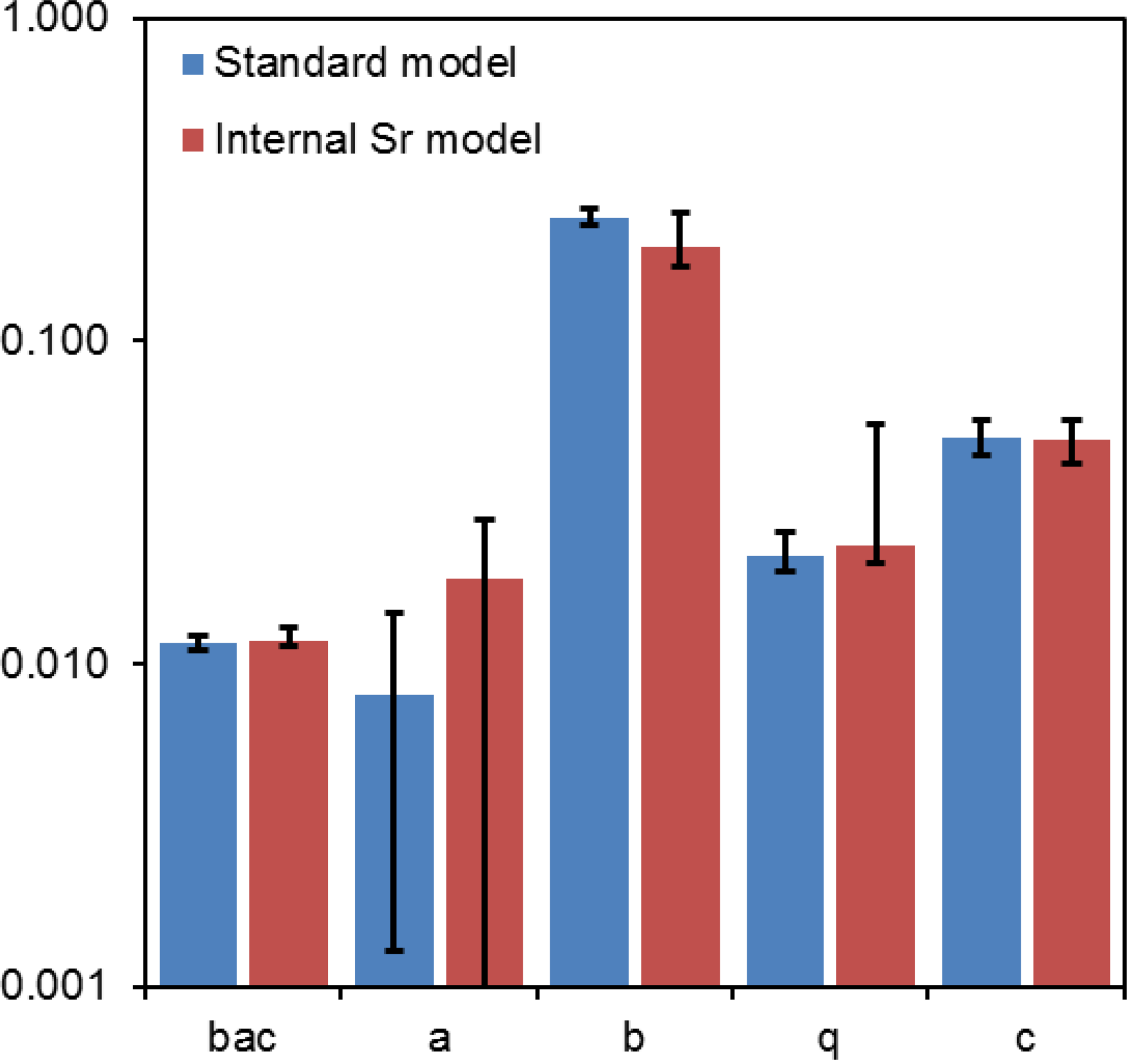
Exploration of model parameter uncertainties by MC simulation which included random variability in dose rate estimates and aberration counts. The bars represent best-fit parameter values for the standard (blue) and internal Sr models (red). Parameter interpretations and units are provided in Table 1. Error bars represent 95% CIs. The best-fit parameter values for the standard model were: *bac* = 0.0117 (95% CI: 0.0110, 0.0122), *a* = 0.0080 (0.0013, 0.0143) year/Gy, *b* = 0.242 (0.228, 0.259), *q* = 0.0216 (0.0193, 0.0255) Gy/year, *c* = 0.0506 (0.0440, 0.0570) year^-1^. For the internal Sr model they were: *bac* = 0.0117 (0.0114, 0.0130), *a* = 0.0183 (0, 0.0279) year/Gy, *b* = 0.197 (0.169, 0.252), *q* = 0.0234 (0.0205, 0.0551) Gy/year, *c* = 0.0498 (0.0415, 0.0570) year^-1^. Details are described in the main text.

Visual comparison of the fits from the standard and internal Sr models (Figs 1 and 3) shows that the shape of the radiation response changed because weighting the internal ^90^Sr contribution decreased the differences in radiation levels between the highly contaminated water bodies (Glubokoye, Dalyokoye and Azbuchin lakes and Yanovsku crawl). This occurred because the fraction of the total dose rate which was due to internal radionuclide incorporation differed strongly among locations: e.g. internal exposure contributed only 5% to the total dose rate in Globokoye lake, but >50% in Azbuchin lake and Yanosvky crawl [30]. Since the data from highly contaminated locations were therefore brought closer together on the x-axis in Fig 3 vs. Fig 1, the best-fitting radiation response became closer to linear. This resulted in a larger best-fit value for the TE parameter a from the internal Sr model, compared with the standard model (Fig 4). However, when random variability in dose rate estimates and aberration counts was included in the estimation of model parameter uncertainties, the 95% CI for parameter *a* overlapped zero in the internal Sr model (Fig 4). All other model parameter values and uncertainties were similar in both the standard and internal Sr models (Fig 4).

**Fig 5:**
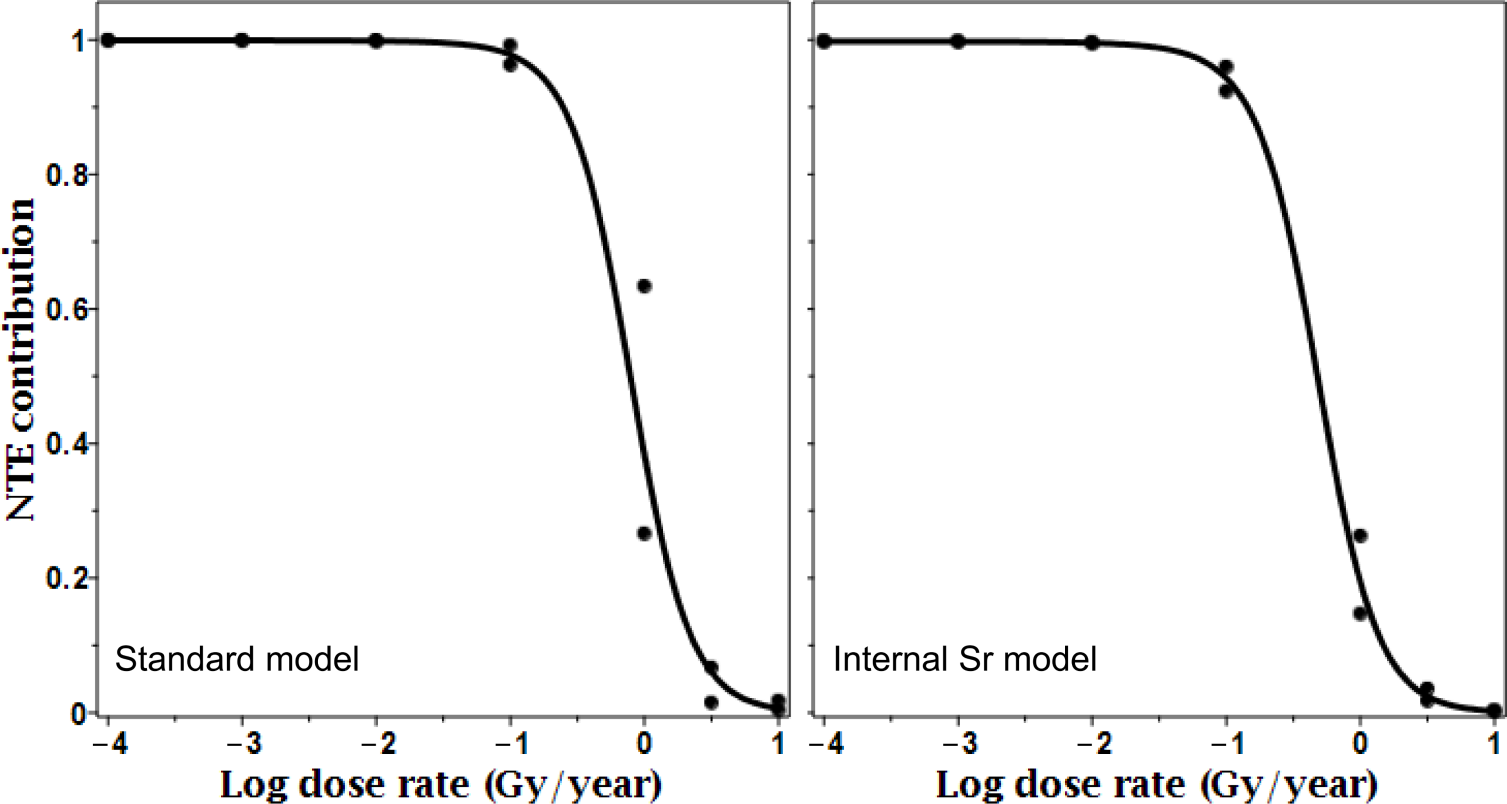
NTE contributions (Eq 11) to the radiation responses predicted by the standard and internal Sr models. Curves represent best-fit results, and symbols show the lower and upper 95% CIs at selected dose rates. The dose rate shown on the x-axis is excess dose rate (above natural background). Details are described in the main text.

As expected based on model assumptions and structure, NTE were particularly important for describing the data at low dose rates (e.g. <1 Gy/year or <100 μGy/h), whereas at higher dose rates their predicted contribution to the radiation response declined (Table 3, Fig 5). This is intuitive because at low dose rates traversals of cell nuclei by ionizing tracks and the TE resulting from such traversals are rare events. Consequently, if the magnitude of the biological effects observed at such dose rates is considerable (as in the data analyzed here), these effects may be caused by NTE.

**Table 3:** NTE contributions (Eq 11) to the radiation responses predicted by the standard and internal Sr models. Excess dose rate represents radiation exposure above natural background.

Excess dose rate (Gy/year)	Lower and upper 95% CIs for NTE contribution			
	Standard model		Internal SR model	
0.0001	0.999	1.000	0.997	0.998
0.001	0.999	1.000	0.997	0.998
0.01	0.997	0.999	0.994	0.997
0.1	0.963	0.992	0.924	0.960
1.0	0.266	0.634	0.147	0.263
3.16	0.015	0.067	0.017	0.036
10.0	0.004	0.018	0.002	0.004

**Fig 6:**
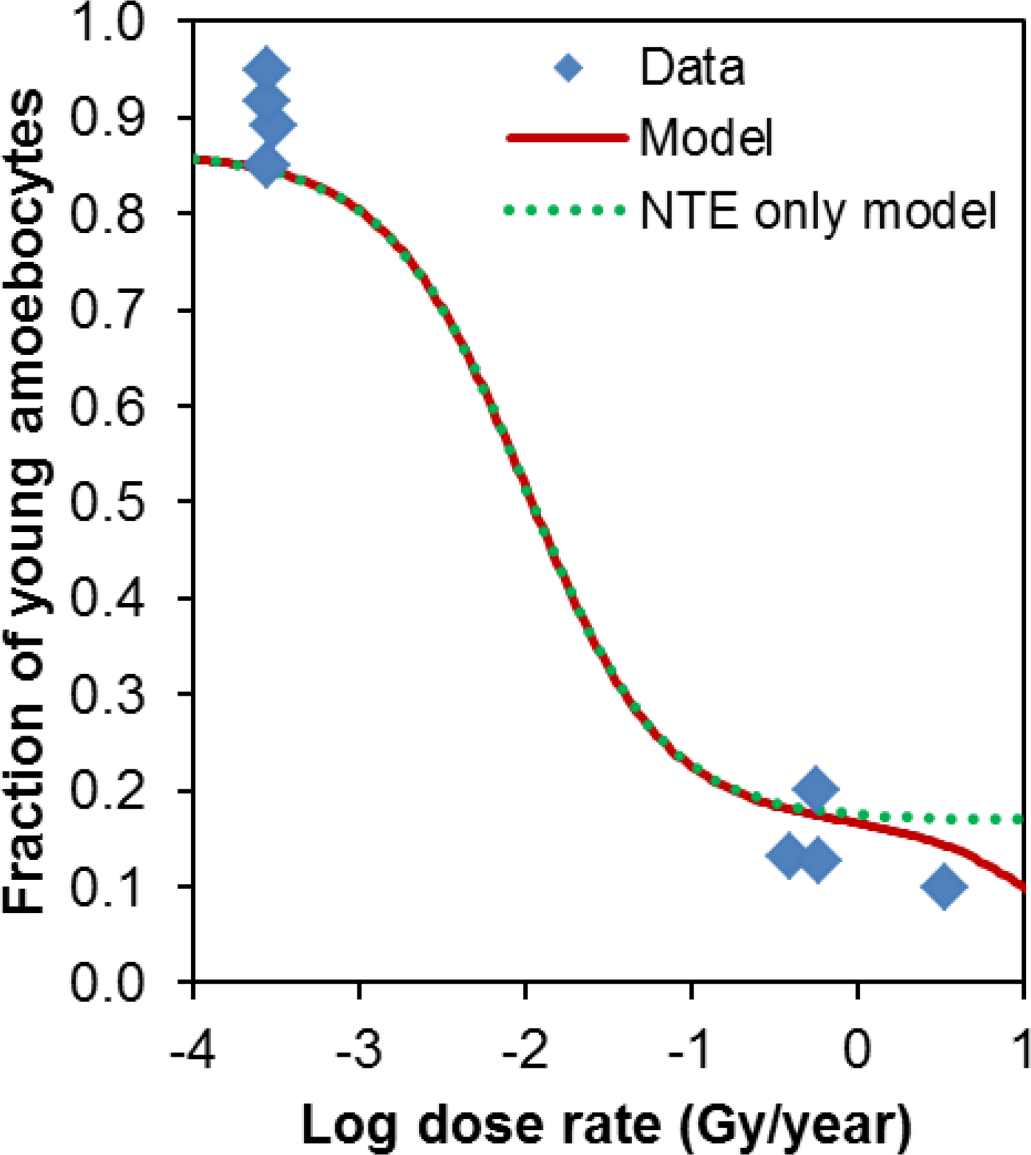
Application of the best-fit radiation response (red curve) produced using the standard model on chromosomal aberration data in snail embryos to a different data set: The fraction of young amoebocytes in the haemolymph of adult snails (blue symbols). Details of this calculation are described in the main text. The NTE-only model curve produced by setting the TE parameter *a* to zero is shown in green.

The effects of radioactive contamination were of course unlikely to be limited to snail embryos–adults were likely to be affected as well. Moreover, if the underlying types of radiation-induced damage and signaling are similar in embryos and adults, then the dose response shapes may also be similar. To investigate this possibility, we applied the best-fit radiation response produced using the standard model on chromosomal aberration data in snail embryos to a different data set: the fraction of young amoebocytes in the haemolymph of adult snails [31] (Fig 6). As described in Materials and Methods, this calculation involved only one adjustable parameter (*s*), which scaled the dose response from the embryo data to the adult data, but did not alter its shape. The best-fit value of *s* was 12.65, and the fitted curve was visually consistent with the data (Fig 6). This result provides some evidence that radioactive contamination affected different snail life stages in similar ways.

## Discussion

We conducted a mechanistically-motivated quantitative analysis of the effects of chronic irradiation from Chernobyl nuclear-power plant accident fallout on natural populations of a common aquatic invertebrate: the pond snail *Lymnaea stagnalis*. The analyzed data sets on chromosomal aberrations in snail embryo cells and on the composition of adult snail haemolymph were presented graphically in a publication by Gudkov et al. [31]. We reconstructed the data by digitizing the published graphs and using additional information reported in references [30, 31]. Certainly this reconstruction was not exact, but the similarity of model parameter estimates obtained by fitting unperturbed (Table 2) or randomly perturbed data (Fig 4) suggested that random errors in reconstruction of the data set were unlikely to strongly affect model parameter estimates and conclusions. We also believe that the following strengths of the data compensated for its limitations: (1) The data sets were very large– 307,540 snail embryo cells and 96,060 adult snail haemolymph cells were analyzed. (2) The measured endpoints–chromosomal aberrations and haematological effects–have relevance for radiation protection. (3) The range of studied dose rates was very broad–approximately 4 orders of magnitude. (4) The study involved multiple locations (8 different water bodies) and spanned multiple generations.

These data showed a non-linear radiation response (Fig 1). The robustness of the response, particularly at low dose rates (< 1 Gy/year) where TE resulting from traversal of nuclear DNA by ionizing tracks are likely to be rare events, suggested involvement of NTE. Our detailed quantitative analysis using a mathematical model which included both TE and NTE terms supported this hypothesis: accounting for NTE was very important for describing the data, whereas a model without NTE performed very poorly (Fig 1, Table 2). NTE were needed for reproducing the non-linear radiation response shape at low dose rates, whereas a TE-only model predicted a linear response which was inconsistent with the data.

The cell signaling pathways responsible for radiation-induced NTE are actively studied but the complex mechanisms involved remain incompletely understood [20, 24, 25, 28, 43–45]. For example, when only some (not all) cells in an organ or organism are damaged by radiation, NTE can convert the entire organism into a stressed state. Such a state, particularly if it persists during chronic radiation exposure, can result in different consequences: oxidative stress and DNA damage levels can increase, but antioxidants and DNA repair machinery can be induced as well [2, 46, 47]. This physiological shift can lead to increased mutagenesis and carcinogenesis, but may also increase the organism’s resistance to massive radiation insults (e.g. to a large acute dose) [25, 26, 48]. Some evidence for the latter in *L*. *stagnalis* is reported by Golubev et al. [32]: snails with higher levels of internal radioactive contamination (i.e. those collected from the most contaminated water bodies) tended to survive longer after acute 500 Gy exposure, than snails with lower contamination levels.

Our analysis provided some evidence that different radionuclides may have different effectiveness in inducing NTE. For example, the best-fit weighting factor for internally accumulated ^90^Sr was 25 (95% CI: 13, 46). ^90^Sr and its decay product ^90^Y are almost pure β emitters. Although the DNA damaging and cytotoxic potencies of β-particles are not as high as those of α-particles, the high importance of ^90^Sr (rather than some α-emitting radionuclides) in *L*. *stagnalis* is plausible because molluscs accumulate ^90^Sr in their shells [49]. In other words, ^90^Sr may be highly relevant for molluscs not only because of its radioactivity, but because of the combination of its radioactivity with its chemical properties, which result in its long-term incorporation into mollusk tissues.

Interactions of radiation with other environmental factors (e.g. water temperature, conductivity, pH, etc), and with chemical pollutants and/or pathogens, could in principle affect chromosomal aberration yields in the studied snails. Variations in snail population structure (e.g. age/stage distributions) at different times or locations also could confound the effects of radiation. Unfortunately, such factors could not be investigated using the analyzed data from Gudkov et al. [30, 31]. However, it is unlikely that they could have distorted the main conclusions of our analysis because differences in measured endpoints (e.g. chromosomal aberrations) between locations (water bodies) with different environmental conditions were not as large as the differences between locations with background, low or high radiation dose rates (Fig 1). In other words, chromosomal aberrations were consistently rare in snail embryos from all water bodies with low radiation levels and consistently much more common in embryos from heavily contaminated water bodies, suggesting that radiation was the dominant cause of the differences in chromosomal aberration frequency.

Overall, the data from Gudkov et al. [30, 31] and our mechanistically-motivated analysis of these data suggested that even though Chernobyl fallout may not have affected the abundance of aquatic molluscs [50], it affected *L*. *stagnalis* on a physiological level. Most of the effects at low dose rates (<1 Gy/year) were likely to be caused by NTE (Table 3, Fig 5). These results imply that NTE may be important for radiation protection for chronic low dose rate exposures, and that developing strategies to disrupt/modulate NTE may be a promising approach to mitigate deleterious chronic radiation effects.

## Supporting information

S1 Data Data used for radiation response modeling. (XLSX)
